# Phylogenomic Analysis of *Salmonella* *enterica* subsp. *enterica* Serovar Bovismorbificans from Clinical and Food Samples Using Whole Genome Wide Core Genes and *kmer* Binning Methods to Identify Two Distinct Polyphyletic Genome Pathotypes

**DOI:** 10.3390/microorganisms10061199

**Published:** 2022-06-11

**Authors:** Gopal R. Gopinath, Hyein Jang, Junia Jean-Gilles Beaubrun, Jayanthi Gangiredla, Mark K. Mammel, Andrea Müller, Sandeep Tamber, Isha R. Patel, Laura Ewing, Leah M. Weinstein, Caroline Z. Wang, Samantha Finkelstein, Flavia Negrete, Tim Muruvanda, Marc Allard, Donald C. Sockett, Franco Pagotto, Ben D. Tall, Roger Stephan

**Affiliations:** 1 Center of Food Safety and Applied Nutrition, U.S. Food and Drug Administration, Laurel, MD 20708, USA; hyein.jang@fda.hhs.gov (H.J.); junia.jean-gillesbeaubrun.civ@mail.mil (J.J.-G.B.); jayanthi.gangiredla@fda.hhs.gov (J.G.); mark.mammel@fda.hhs.gov (M.K.M.); isha.patel@fda.hhs.gov (I.R.P.); laura.ewing-peeples@fda.hhs.gov (L.E.); lmweinstein1@gmail.com (L.M.W.); caroline.z.wang@duke.edu (C.Z.W.); sfinkel6@terpmail.umd.edu (S.F.); flavianegrete@yahoo.com (F.N.); shakee2326@aol.com (B.D.T.); 2 Biological Analysis Division, Public Health Command Europe Laboratory Sciences, Room 102, Bldg 3810, Kirchberg Kaserne, RP 66849 Landstuhl, Germany; 3 Institute for Food Safety and Hygiene, University of Zurich, CH-8057 Zurich, Switzerland; andreamonika.mueller@uzh.ch (A.M.); stephanr@fsafety.uzh.ch (R.S.); 4 Food Directorate, Bureau of Microbial Hazards/Health Canada, Ottawa, ON K1A 0K9, Canada; sandeep.tamber@canada.ca (S.T.); franco.pagotto@canada.ca (F.P.); 5 Center of Food Safety and Applied Nutrition, U.S. Food and Drug Administration, College Park, MD 20740, USA; tim.muruvanda@fda.hhs.gov (T.M.); marc.allard@fda.hhs.gov (M.A.); 6 Wisconsin Veterinary Diagnostic Laboratory, University of Wisconsin-Madison, Madison, WI 53706, USA; donald.sockett@wvdl.wisc.edu

**Keywords:** *Salmonella* Bovismorbificans, phylogenomics, plasmids, phages, virulence factors

## Abstract

*Salmonella enterica* subsp. *enterica* serovar Bovismorbificans has caused multiple outbreaks involving the consumption of produce, hummus, and processed meat products worldwide. To elucidate the intra-serovar genomic structure of *S*. Bovismorbificans, a core-genome analysis with 2690 loci (based on 150 complete genomes representing *Salmonella enterica* serovars developed as part of this study) and a *k-mer*-binning based strategy were carried out on 95 whole genome sequencing (WGS) assemblies from Swiss, Canadian, and USA collections of *S*. Bovismorbificans strains from foodborne infections. Data mining of a digital DNA tiling array of legacy SARA and SARB strains was conducted to identify near-neighbors of *S.* Bovismorbificans. The core genome analysis and the *k-mer*-binning methods identified two polyphyletic clusters, each with emerging evolutionary properties. Four STs (2640, 142, 1499, and 377), which constituted the majority of the publicly available WGS datasets from >260 strains analyzed by *k-mer*-binning based strategy, contained a conserved core genome backbone with a different evolutionary lineage as compared to strains comprising the other cluster (ST150). In addition, the assortment of genotypic features contributing to pathogenesis and persistence, such as antimicrobial resistance, prophage, plasmid, and virulence factor genes, were assessed to understand the emerging characteristics of this serovar that are relevant clinically and for food safety concerns. The phylogenomic profiling of polyphyletic *S*. Bovismorbificans in this study corresponds to intra-serovar variations observed in *S.* Napoli and *S.* Newport serovars using similar high-resolution genomic profiling approaches and contributes to the understanding of the evolution and sequence divergence of foodborne *Salmonellae*. These intra-serovar differences may have to be thoroughly understood for the accurate classification of foodborne *Salmonella* strains needed for the uniform development of future food safety mitigation strategies.

## 1. Introduction

*Salmonella* species are one of the leading causes of foodborne outbreaks and systemic infections worldwide [1] and in the USA [2], leading to thousands of deaths every year. Human infections with *Salmonella enterica* subspecies *enterica* serovar Bovismorbificans are rare compared with other *Salmonella* serovars such as Typhimurium or Enteritidis. However, *S.* Bovismorbificans have increasingly been reported as an emerging human pathogen causing foodborne illnesses in Asia [3] and in the western hemisphere. The molecular epidemiology of a sprout-borne outbreak of *S.* Bovismorbificans [4,5] in Finland had been reported earlier. Most foodborne outbreaks associated with *S.* Bovismorbificans reported in Europe, the USA, and Canada has been traced to pork products, lettuce, hummus, and sprouts [6,7,8]. The first genomes of *S.* Bovismorbificans were generated from a Malawian bacteremia case and UK veterinary samples belonging to Sequence Type (ST)142 (ST142) including that of a virulence plasmid pVirBov from clinical strain 3114 [9]. More ST142 virulent strains were isolated from outbreaks associated with uncooked ham products in the Netherlands [10]. Interestingly, these strains possessed a 5.1 kb col156 plasmid. The draft genomes from food and clinical strains from a 2011 outbreak of *S.* Bovismorbificans in Washington, DC, USA isolated from contaminated hummus samples were made available [11,12,13]. These strains were typed as ST377 and were phylogenetically distinct from other USA food and environmental strains reported independently [14] belonging to ST150. These reports implicated ST142 and ST377 as the predominant sequence types of *S.* Bovismorbificans contaminating the food supply and causing foodborne illnesses in the USA and Europe. In 2018, the Wisconsin Veterinary Diagnostic Laboratory (WVDL) isolated *S.* Bovismorbificans strains from environmental samples that were collected from a Minnesota dairy calf production facility. During this time, the dairy industry experienced an increased mortality event in 25% of dairy calves at 1–3 weeks of age caused by *S.* Bovismorbificans (personal communication, DCS). Environmental sampling also showed the presence of *S.* Bovismorbificans. This finding prompted the hypothesis that *S.* Bovismorbificans may be persistent in this dairy production environment. A study conducted [15] on the impact of *S.* Bovismorbificans along the food supply chain in Hungary investigated contaminated food production environments, animals, foods of animal origin, and humans and provided evidence that *S.* Bovismorbificans is less invasive to host animals than *S.* Enteritidis but may colonize and persist in several animal species leading to contamination of meat.

There is considerable interest in identifying underlying bacterial genomic attributes related to the increased transmission of minor serovars such as *S.* Bovismorbificans in humans and within the animal food supply chains. The diversity of virulence mechanisms, differences in the genomic features, and emergent sources/niches are increasingly recognized as major contributors to the success of *Salmonella* as a major foodborne pathogenic group [16]. Genome-wide variations of different groups within the serovars of *S. enterica* have been extensively studied using whole genome sequencing (WGS) datasets. A high number of polyphyletic lineages in many serovars have been predicted using MLST and genome-wide SNP profiling methods [17,18,19,20,21,22]. For example, phylogenetic analysis of 156 WGS datasets from 78 serovars using about 120,000 whole genome SNPs present in at least 95% of the strains identified the presence of polyphyletic lineages in a handful of serovars [19]. Similar WGS-based methods were applied to analyze *S.* Newport [20] and *S.* Napoli [21] strains. In the present study, 95 strains of *S*. Bovismorbificans were collected from the USA, Switzerland, and Canada from various types of sources and years. Phylogenomic sequence analysis of the strains was conducted using a core genome schema developed using 150 complete genomes. This resulted in a scalable ad hoc bioinformatic workflow to identify the core genome among *S.* Bovismorbificans, which resulted in the identification of two distinct polyphyletic groups within the serovar with significant divergence in their core gene loci. In addition, a *k-mer*-binning method and data mining of digital DNA tiling array profiles were applied in parallel to illustrate this evolutionary relationship among the sub-groups of this important emerging *Salmonella* pathogen. We suggest that this approach can be applied to predict and annotate the emerging virulence properties of under-surveyed minor serovars of *Salmonella* with the potential to cause sporadic foodborne outbreaks.

## 2. Materials and Methods

### 2.1. Bacterial Strains

A total of 95 strains of *S.* Bovismorbificans isolated from Switzerland, the USA, and Canada were analyzed in this study. The collection represents clinical, food, feed, animal, and environmental strains, and metadata for these strains including country, year of isolation, and genomic attributes. *S.* Bovismorbificans strains collected from Switzerland, the USA, and Canada were provided by University of Zurich, University of Wisconsin-Madison, and Health Canada, respectively. In addition to the newly sequenced 81 strains, genomes from 14 *S*. Bovismorbificans genomes, which were associated with several hummus USA outbreaks, were included [7,11,14]. For comparative analysis, a scaffolded genome from the clinical strain (3114) from Malawi [9], an environmental strain (CIES13) from Mexico, and 25 more *S*. Bovismorbificans genomes were downloaded from NCBI. All strains were stored at −80 °C in Trypticase soy broth (TSB; BBL, Cockeysville, MD, USA) supplemented with 50% glycerol. All strains were serotyped and identified according to the White–Kauffmann–Le Minor scheme by slide agglutination [23], USA strains were also serotyped by PCR analysis, as described earlier [24]. Genome assemblies from the strains were used to confirm each strain’s serotype using the SeqSero2 v1.0.2 [25] application on CFSAN’s GalaxyTrakr [26] at https://galaxytrakr.org/ (accessed on 5 January 2022).

### 2.2. Genomic DNA Preparation

Frozen stocks of each strain were streaked onto Xylose-lysine-tergitol 4 (XLT4; BBL, Cockeysville, MD, USA) agar plates and cultured at 37 °C for overnight. A single typical colony (black or black-centered) of each strain on XLT4 was inoculated into 5 mL of TSB supplemented with 1% NaCl (TSBS), and then incubated at 37 °C for 20 h with shaking at 150 rpm. Genomic DNA was extracted from the overnight cultures using a Qiagen QIACube instrument and its automated technology (QIAGEN Sciences, Germantown, MD, USA) according to the manufacturer’s instructions. Typical yields of the purified genomic DNA are 5–50 µg from a final elution volume of 200 µL, and duplicated DNA samples were prepared for WGS and microarray experiments. Each strain’s DNA was quantified using a Qubit dsDNA BR assay kit (Invitrogen, Thermo Fisher Scientific, Wilmington, DE, USA) and Qubit 2.0 fluorometer (Life Technologies, Grand Island, NY, USA).

### 2.3. Whole Genome Sequencing (WGS), Assembly, and Annotation

Eighty-one strains were obtained from Switzerland, the USA, and Canada, and representing clinical (69), food (9), feed (1), animal (1), and environment (1) strains (isolated during 1984–1989 and 2011–2018) were sequenced in this study. Each DNA sample prepared as described above was diluted in nuclease-free deionized water (molecular biology grade, Thermo Fisher Scientific, Waltham, MA, USA) to achieve a final concentration of 0.2 ng/μL. WGS libraries of these strains (50× coverage) were constructed using the Nextera XT DNA sample preparation kit (Illumina, San Diego, CA, USA). Genome sequencing was performed on a Miseq platform using either 500 or 600 cycles of paired-end reads (Illumina). FastQ datasets (raw reads) were trimmed for removal of adaptor sequences and for quality control purposes, and de novo assembled using CLC Genomics Workbench version 9.0 (CLC Bio, Aarhus, Denmark). The genomes were independently annotated using the Rapid Annotation Subsystems Technology (RAST) annotation server [27] for quality control and accuracy. The genome sizes, the number of coding sequences (CDS), ST assignments, NCBI BioSample ID, and accession numbers of these assemblies are shown in Table 1. PacBio RSII platform was used following the manufacturer’s protocols to generate a complete genome of a 93kb plasmid from Sal610. All the assemblies (Table 1) along with the prokaryotic genome annotation pipeline (PGAP) annotations [28] were deposited into NCBI’s GenBank and used in the subsequent analyses as needed. The datasets were released to the public through submission to NCBI under the FDA-CFSAN’s GenomeTrakr *Salmonella enterica* BioProject PRJNA378379, which is part of the CFSAN’s foodborne pathogen research umbrella project PRJNA186875 at NCBI [29].

### 2.4. Identification of Whole-Genome Core Genes and High-Resolution Phylogenomic Analysis

A total of 645 complete genomes (Supplemental File S1) of *S. enterica* representing 150 serovars were downloaded from NCBI’s Genome database (20 February 2020) to generate a large local BLAST database. Some *Salmonella* serovars such as *S.* Typhimurium and *S.* Enteritidis were overrepresented in this pool, while most others had few or single completed genomes. A shorter list of representative genomes of the 150 serovars was randomly chosen for subsequent analysis in this bioinformatic workflow. CDS (4606) annotations of *S. enterica* serovar Typhimurium LT2 genome (NC_003197) were downloaded from GenBank and used as a query in the BLAST analysis at 50, 90, and 95% identity levels. In-house Perl and Python scripts (available upon request) were used to parse the BLAST outputs and to create a SNP-finding workflow. Manual curation of the resulting data matrix with homology search and alignment contributed to the generation of a whole genome core genes (wg-core) set of 2690 loci representing a conserved genomic backbone of representative genomes for 150 complete *Salmonella* serovars.

WGS assemblies from *S*. Bovismorbificans strains from this study and external sources (Table 1) were queried with the wg-core gene set to identify conserved backbone genes. A SNP data matrix consisting of homologs of wg-core genes in the evaluated *S.* Bovismorbificans strains with at least one allele in each of the 2512 out of 2690 loci was created (Supplemental File S2 also available from https://github.com/gopal-gopinath/S.bovismorbificans-SNP-matrix1 (last accessed 6 May 2022). The evolutionary distances were computed using the Maximum Composite Likelihood method [30] and the phylogenetic tree was built based on the neighbor-joining method [31] as implemented on the MEGA X phylogenetic suite [32] and UPGMA algorithm implemented in SplitsTree version 5 [33]. The SNP matrices were curated for quality to remove missing genes and partial gene sequences represented by gaps after multiple alignments as part of the routine quality control. The genome-wide *k-mer*-binning analysis was carried out by generating a Jaccard similarity matrix with *k-mer* content using in-house scripts. For each genome, a list of all *k-mers* (*k* = 30) present in the sequences was stored. Each pair of genomes was compared for *k-mer* content to derive the Jaccard similarity score defined as the size of the intersection divided by the size of the union of the *k-mer* sets.

### 2.5. Data Mining of a DNA Tiling Microarray Database for Genomic Comparisons with Legacy Strain Collections

The FDA *Salmonella* custom high-density Affymetrix DNA microarray platform was used, as previously described [34,35]. An 8 μg aliquot of purified genomic DNA was fragmented by incubation at 37 °C for 10 min in a 50 μL reaction containing 1× One-Phor-All Plus Buffer [Tris, Magnesium and Potassium acetate (Ratios 1:1:5)] and 0.1 units DNase I (GE Healthcare, Pittsburg, PA, USA). Following fragmentation, the DNA was labeled at the 3′-end using 1 mM biotin-11-ddATP (PerkinElmer NEL508, Waltham, MA, USA), 5X terminal transferase buffer (Promega, Madison, WI, USA), and 60 units of terminal transferase enzyme (Promega), as described earlier. The genomic DNA samples were hybridized following the Affymetrix GeneChip Expression Analysis Technical Manual (Affymetrix, Santa Clara, CA, USA, 2014), washed in the Affymetrix FS-450 fluidics station (Affymetrix, Santa Clara, CA, USA), and scanned using software of the Affymetrix GeneChip Command Console (AGCC) Scanner 3000. Reagents used in hybridization, washing, and staining were prepared according to the Affymetrix GeneChip Expression Analysis Technical Manual [36]. For microarray data analysis, a probe set intensity for each allele represented on the microarray chip were assessed using the Robust MultiArray Averaging (RMA) function in the Affymetrix package of R-Bioconductor [37]. 

### 2.6. Characterization of Multi-Locus Sequence Typing (MLST) and Antimicrobial Resistance Gene (AMR) Patterns

Sequence types of all *S.* Bovismorbificans genomes used in this study were determined using the MLST schema [17,38] mplemented on the Center for Genomic Epidemiology (CGE) web servers (http://cge.cbs.dtu.dk/services/MLST, accessed on 6 August 2021). For serological confirmation and antimicrobial resistance gene analyses, genome assemblies (FASTA files) were uploaded to the CFSAN’s GalaxyTrakr SeqSero and AMRFinderPlus tool, respectively (https://galaxytrakr.org/root/login?redirect=%2F, accessed on 6 August 2021). CFSAN’s Galaxy GenomeTrakr AMRFinderPlus tool scans each genome against the accompanying database, which is designed to find acquired antimicrobial, biocide, heat, acid, and metal resistance genes in bacterial protein or assembled nucleotide sequences, as well as known point mutations for several taxa [39]. The classes of antimicrobials that the CFSAN Galaxy GenomeTrakr AMRFinderPlus tool identifies include aminoglycoside, beta-lactams, bleomycin, colistin, fosfomycin, fusidic acid, glycopeptide, nitroimidazole, oxazolidinone, phenicol, quaternary ammonium, quinolone, rifamycin, streptogramin, sulfonamide, tetracycline, and trimethoprim.

### 2.7. Identification of Salmonella Pathogenicity Islands (SPIs), Plasmids, and Prophage Genomic Regions and Virulence Factors

pVIRBov, a virulence plasmid from *S.* Bovismorbificans strains 3114 [9], was downloaded (Acc. No.: HF969016) from NCBI. pVIRBov sequences were used to identify homologous plasmid sequences in the WGS datasets generated from this study (Table 1). For this step, a local database of *S*. Bovismorbificans strains was first created for BLAST analysis as needed. The stringent parameters used for the BLAST analysis (percent identity: 50%, best hit score: 0.05, best hit overhang: 0.25, e-value: 1 × 10^−10^) were chosen to minimize both alignment errors and random mutations for screening the WGS database. In-house Python and Perl scripts were then used to parse the BLAST output and identify homologous sequences of 111 total pSal610/pVIRBov CDS in 95 genomes of *S.* Bovismorbificans strains. BRIG 0.95 software [40] was used for the visualization of plasmid sequence comparisons. Assembled genomes of 34 *S*. Bovismorbificans strains representative of the various STs were submitted to the CGE SPIFinder (https://cge.cbs.dtu.dk/services/SPIFinder, accessed on 6 August 2021) to detect *Salmonella* pathogenicity islands (SPIs). The default parameters with threshold for ID of 95% and minimum length at 60% for SPIFinder 1.0 tool settings were used to minimize noise and to eliminate any gene fragments, which enables the detection of genes in the start or end of contigs. The CGE PlasmidFinder tool [41] available at https://cge.cbs.dtu.dk/services/PlasmidFinder (accessed on 6 August 2021) was employed to detect replicon-associated plasmid sequences using the *Enterobacteriaceae* as the default settings: 95% minimal identity and 60% minimal coverage. Prophage genomic regions were identified using the PHASTER (Phage Search Tool Enhanced Release) web server (https://phaster.ca, accessed on 6 August 2021). Upon uploading to the PHASTER pipeline, putative phage and prophage regions (i.e., incomplete, questionable, and intact prophage regions) were identified through examination of prophage genes and comparison of predicted proteins against PHASTER’s complete prophage databases [42]. The SEED server was routinely used for sequence comparisons and datamining functional annotations [43]. Prophage sequences from the *S.* Bovismorbificans WGS datasets were identified using the *Salmonella* phage RE-2010 (GenBank Accn#: HM770079). NCBI Genome Tree for *S.* Bovismorbificans at https://www.ncbi.nlm.nih.gov/genome/tree/152? (accessed on 6 September 2021) was used to identify nearest neighbors for *S.* Bovismorbificans.

## 3. Results and Discussion

### 3.1. Genome Sequencing, Assembly and MLST Typing

The *S*. Bovismorbificans genomes in this study ranged from 4.5 to 5.0 MB. A complete genome of a 93 kb virulence plasmid from strain Sal610 was generated (Accession No. #: CP076746; Table 1). The shared *S.* Bovismorbificans and *S*. Hindmarsh immunogenic cluster signature from SeqSero was 8:r:1,5. The MLST tool on the CGE server grouped the *S.* Bovismorbificans strains into five different STs: 142, 2640, 377, 1499, and 150 (Supplemental Table S1). Nine out of 95 genomes were typed as ST150 and the rest were identified as ST142 or one of the highly similar STs: 2640, 377, and 1499 (Table 1) that differ from each other in one of the seven MLST loci. ST150 has a highly divergent allelic pattern (Supplemental File S1), where all seven alleles display significant differences from the ST142 strains and the other three STs. Comparative sequence analysis on the SEED server and local BLAST analysis of strain sal610 (ST377), Swiss strains (ST142, ST 1499, and ST2650), and strains sal676-681, N16_1558 and M18_12182 (ST150) consistently pointed to a high degree of genomic differences (data not shown) among these groups of strains. These preliminary analyses suggested extensive genome-wide sequence differences among the *S*. Bovismorbificans strains that were not fully captured by the conventional techniques consistent with the limitations of such approaches observed in other situations [44].

### 3.2. Data Mining of DNA Tiling Digital Hybridization Profiles Identified Distinct Genomic Backbone Differences

Digital profiles from DNA tiling microarray hybridization experiments were created for representative strains of different STs and isolation sources using the FDA SEEC microarray platform. Initial data mining of an accompanying database made up of digital hybridization profiles of thousands of *Salmonella* strains sourced from past surveillance efforts and institutional collections suggested nucleotide diversity in the gene content of the queried genomes. For example, the sequence diversity of genes harvested from digital hybridization experiments of previously reported hummus-outbreak and surveillance *S.* Bovismorbificans strains [11,14] showed that the hummus strains were more closely related to *S.* Typhimurium (SARA2) and *S.* Muenchen consisting of SARA63-66 [38,45], while the surveillance strains (ST150) clustered as a distinct, independent group (Supplemental Figure S1A). Additionally, clinical Swiss *S.* Bovismorbificans strains belonging to ST142, ST2640, and ST1499 were more related to one another than to the ST150 strains. When the digital profiles of representative strains from different STs originally reported [8] and included in this study were queried against this database, they sorted between these two clusters (Supplemental Figure S1B), as expected. Previous reports [17,35] had pointed to significant sequence variations within STs using techniques ranging from PFGE to WGS to a limited number of genes used for clustering. As foodborne outbreaks of minor, zoonotic serovars such as *S.* Bovismorbificans are being reported to cause illness in humans [8,9] by entering the food-supply chain, a detailed understanding of the genomic differences between the lineages that constitute *S*. Bovismorbificans and other serovars not currently on the top list of public health agencies becomes important for risk assessment, import control, and the development of prevention measures for efficient food safety and public health outbreak management.

### 3.3. Development of a Workflow for Core Genome Analysis Based on Complete Genomes from 150 S. enterica Serovars

We started with 645 complete and fully annotated genomes representing around 150 serovars that were obtained from the publicly available NCBI Genome database (Supplemental File S1). These sequences were combined with the new WGS assemblies of *S.* Bovismorbificans generated from this study (Table 1) to create a large BLAST database. When 4606 *S.* Typhimurium LT2 chromosomal genes were tested against this database at 50, 90, and 95% similarity levels, it yielded 1900 to 3400 chromosomal genes. After initial screening, 2830 genes at 90% similarity level were selected for manual curation to remove loci with alignment gaps, redundant annotations, and sequence quality errors. 2690 LT2 gene loci that contained at least one allelic difference in at least 90% of the genomes were selected as the ‘wg-core gene’ set for our phylogenomic studies. This list included the seven MLST housekeeping gene sequences and all the *S*. Typhimurium LT2 loci used as a reference, which were annotated and available in Supplemental File S6. The wg-core gene schema was tested for accuracy and reproducibility on genomes from an in-house collection of new and published strains (sub-sets of genomes from the data matrix presented in Supplemental File S2) prior to its application on the complete *S.* Bovismorbificans dataset from this study. It must be noted that this schema was tested only on the listed serovars of *S. enterica* subsp. *enterica.* Furthermore, extensive analysis is needed to evaluate the performance of this schema against the genome assemblies of other *S. enterica* sub-species. A broader comparison of the loci from this schema with genome-wide MLST markers in Enterobase [46], various bioinformatic methods used in genomic epidemiology [47] and SISTR database [48] is also necessary for developing a unified wg-core MLST marker dataset.

### 3.4. Genome Pathotyping of S. Bovismorbificans Strains Using Wg-Core Genes and k-Mer-Binning Methods

**Figure 1:**
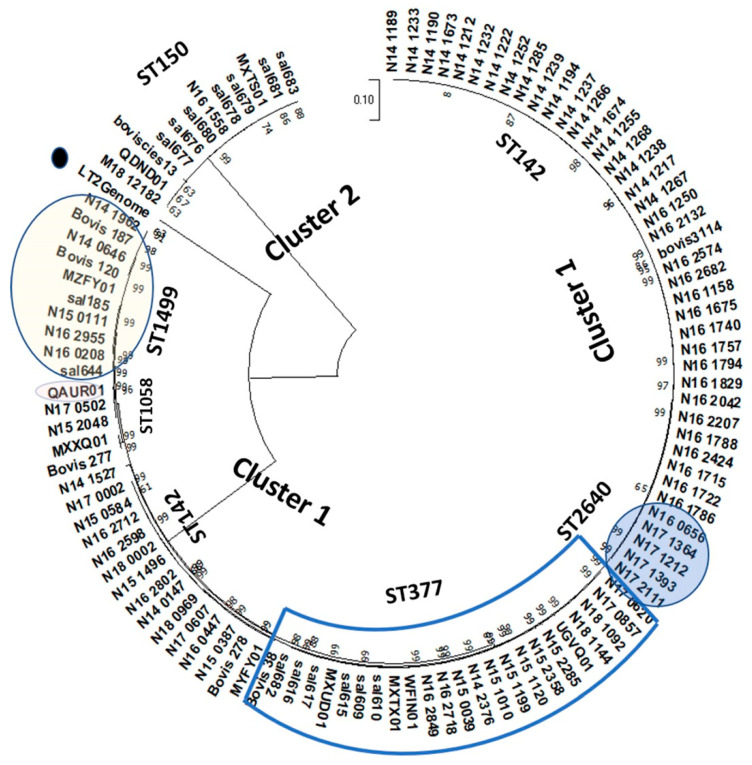
Wg-core gene analysis of 95 *S.* Bovismorbificans identifies two distinct clusters having different genomic backbones. *S.* Bovismorbificans strains were obtained from clinical, animal, feed or food and water or unknown sources isolated during 1984–1989 and 2011–2018 (from this study) and representative strains from NCBI. Cluster analysis was carried out using single nucleotide polymorphisms in 2690 core genes representing conserved backbone, and the phylogenetic tree was developed using the Maximum-Likelihood method [30] available on MEGAX’s phylogenetic suite [32]. *S*. Typhimurium (LT2. single dot) was used as an outlier. Alleles in 48,344 positions were considered across 110 genomes spanning 2650+ out of 2690 core genes, which were considered tested over 500 bootstrapping iterations. The resulting circular tree is shown here. A browsable vertical layout of the tree is available in Supplemental Figure S1. Refer to Supplemental File S2 on the page for the comprehensive allelic data matrix from this analysis.

The 2690 wg-core genes clustered the 95 *S.* Bovismorbificans WGS assemblies into two high-level groups that exhibit distinct genomic differences (Figure 1). The two distinct clusters consisted of a larger cluster (Cluster 1) made up of Swiss, DC-hummus outbreak, and European strains, and a smaller cluster (Cluster 2) consisting of singletons from veterinary, food, and clinical sources from the USA, Switzerland, and Canada. More than 85 *S.* Bovismorbificans genomes from this study are represented in Cluster 1 and phylogenetically sort into the four highly related MLST groups identified earlier (ST142, ST2460, ST377, and ST1499). A ST142 sub-cluster included only Swiss strains from 2014–2016; the 2011 hummus outbreak sub-cluster strains belonging to ST377 grouped together; the ST1499 strains comprised a mixture of Canadian and Swiss strains, and the ST2640 cluster had five Swiss strains. The two polyphyletic genomic pathotypes observed in our study appear to be a serovar-wide phenomenon for *S.* Bovismorbificans when geographically and temporally different external genomes were also included for analysis. For example, seven of the external *S.* Bovismorbificans strains were sorted into Cluster 1 along with these genomes. A publicly available genome assembly, QAUR01 from NCBI, was a single ST1058 strain noted in this study. The five ST groups profiled in Cluster 1 had overlapping MLST allelic formulae with differences in up to three loci in some cases (Supplemental File S3). Our analyses demonstrated a conserved genomic backbone among these strains constituting the bulk of *S.* Bovismorbificans strains with publicly available genomic data (Figure 1). Cluster 2 consisted only of ST150 strains obtained from food, clinical (‘sal’ strains), and veterinary (M18_12182) sources from the US and Canada (‘Bovis’ strains) along with a single Swiss strain, N16_1558. The analysis identified CIES13, MXTS01, and QDND0, sharing a similar genomic backbone with the ST150 strains. The conserved core genomic backbone of the Cluster 1 strains was phylogenetically closer to the *S.* Typhimurium LT2 (reference strain) genome than that of Cluster 2 *S.* Bovismorbificans genomes as determined by this wg-core gene analysis. The conventional serotyping and sequence-based determination of the serovars of these two clusters were based on the immunogenic antigens and their coding sequences, respectively. Even if the *S.* Bovismorbificans strains of these two clusters appear to be sharing similar sequences for the serotyping antigens (based on sequence-based serotyping results from SeqSero2) resulting in the designation of the same serovar, these strains possess highly divergent core genome backbones and appear to be polyphyletic based on the 2690 wg-core gene schema developed as part of this study. These observations with the *S.* Bovismorbificans strains from different regions matched with our initial separation of hummus outbreak and surveillance strains into clusters when compared with the digital DNA tiling microarray profiles of a legacy strain collection (Supplemental Figure S1A,B).

**Figure 2:**
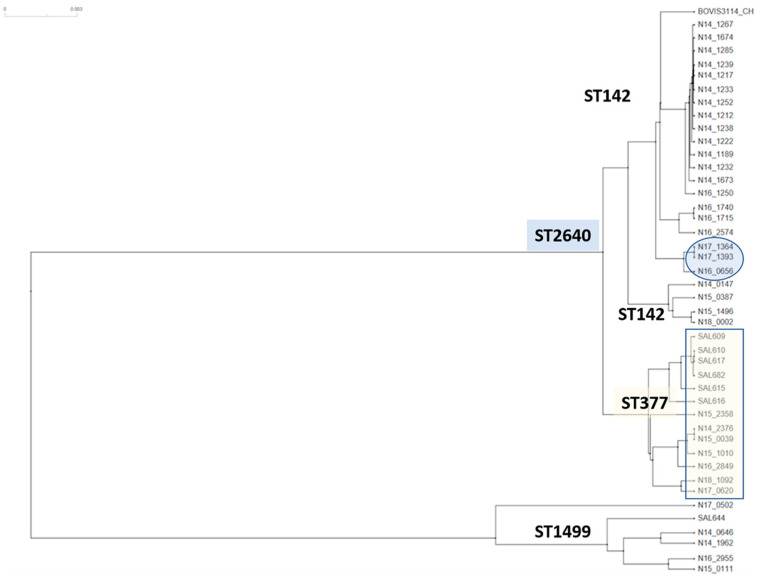
Phylogenetic analysis with wg-core gene SNPs in emerging ST lineages within genome type Cluster 1 of the *S.* Bovismorbificans serovar. The Cluster 1 consisted of 4 major STs and diverged significantly from ST150 strains of Cluster 2. Even among these related strains in Cluster 1, emerging polymorphisms exhibiting different rates of microevolution were observed using the SNP data matrix. UPGMA tree drawn on SPlitsTree 5.0. Refer to Supplemental File S3 for details of SNPs among the strains of different Cluster 1 STs.

The 95 strains from the current study used for this analysis have a large proportion of food and clinical isolates (Table 1) from a Swiss collection. Nevertheless, the architecture of Cluster 1 strains displayed emerging sequence variations characteristic of robust microevolutionary processes, as observed from the emergence of different ST groups. When a sub-set of 35 Cluster 1 genomes was analyzed at higher resolution using wg-core genes, sub-groups of strains with significant SNP differences emerged (Figure 2). Partial quantitation of allelic differences in ST377, ST2640, and ST1499 strains individually in comparison with ST142 strains and micro-evolutionary profiling of the associated core-gene loci was carried out using annotations of reference strain LT2 (Supplemental File S3). For example, the ST1499 group consists of strains demonstrating significant sequence divergence in hundreds of core gene loci when compared with ST142. The core gene loci associated with the ST1499 strains included kinases, transport and membrane proteins, fimbriae, and enzymes involved in various metabolic pathways, which appeared to be undergoing a faster rate of evolutionary change (Supplemental File S3, worksheet 2). The Cluster 2 ST150 strains also predictably exhibited sequence variations in the core-gene loci (Supplemental File S3, worksheet 4). Metadata analysis combined with these phylogenetic clusters also showed that in Switzerland from 2014–2018, *S.* Bovismorbificans strains belonging to four STs: 142, 377, 1499, or 2640 were prevalent at different time points, indicating long-term persistence of *S*. Bovismorbificans strains in the food supply chain. These results suggested that: (i) many Cluster 1 *S.* Bovismorbificans strains belonging to different conventional ST groups displayed a phylogenetically distinct, shared genomic backbone, and (ii) the emergent properties within these sub-groups of spatially and temporally discrete lineages were undergoing robust, quantifiable micro-evolutionary changes. However, robust biological experimentation is required to assess the phenotypic impact of these micro-evolutionary changes.

Manual curation of NCBI Genome Tree for *S.* Bovismorbificans and data mining of the DNA Tiling database from the SEEC microarray platform (Supplemental File S2B) identified *Salmonella* serovars Muenchen and Hindmarsh as the closest neighbors to the strains in Cluster 1, and serovar *S.* Takoradi for strains in Cluster 2. To resolve the nearest neighbors to the *S.* Bovismorbificans clusters that would clarify the differences in the core-gene loci we had observed earlier, we carried out *k-mer*-binning analyses on a global collection of whole genomes from 265 *S.* Bovismorbificans (inclusive of the 95 from this study from Table 1), 60 *S.* Muenchen and nine *S.* Hindmarsh strains (Accession and serovars listed in columns A and B, respectively, in Supplemental File S4). The analysis generated a data-matrix with 330 × 330 datapoints and quantified values of each binary genomic comparison as a *k-mer* ratio were visualized as a heat-map color-coded in a teal blue (proximal) to orange (distal) values (Supplemental File S4, worksheet titled ‘Jaccard Matrix’). Many *S.* Hindmarsh strains were similar to the *S.* Bovismorbificans strains found associated with Cluster 1, strains; however, two—AAOBOO01 (containing “8:r:” formula according to SeqSero) and SRR3710239 (containing the typical “8:r:1,5” formula on SeqSero)—appear to contain a Cluster 2 genomic backbone such as ST150 *S.* Bovismorbificans strains.

**Figure 3:**
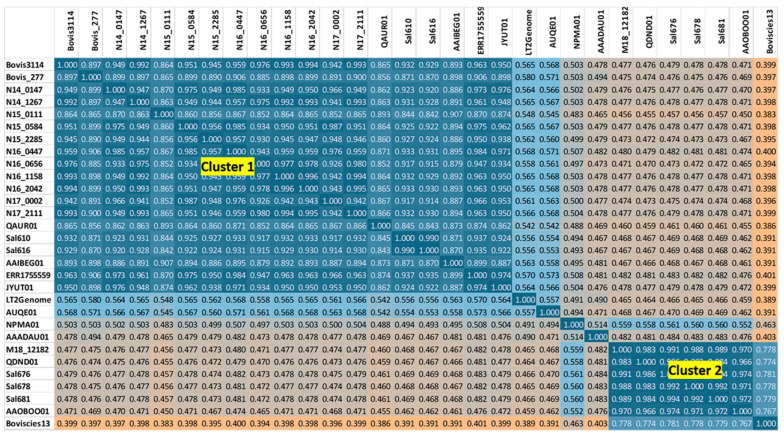
*k-mer*-binning analysis of genomes from *S*. Bovismorbificans and closely related serovars. A snap shot of the Jaccard index from the *k-mer*-binning analysis (*k* = 30) of 330 WGS assemblies dataset from five *Salmonella* serovars was illustrated (Heatmap orange to teal blue = most divergent to most similar). *S*. Typhimurium (LT2Genome) and *S*. Muenchen (AUQE01) are closer to Cluster 1 strains (shown in sky blue), and *S*. Takoradi (NPMA01) is the closest serovar to the ST150 Cluster 2 strains (shown in tan). Distinct genomic pathotypes of the two *S*. Bovismorbificans clusters suggest evolutionarily independent hinging of a single serotyping gene cluster (“8:r:1,5”) in two different lineages of SE with different genomic backbones. The complete phylogenomic analysis with 330 genomes is presented as Supplemental File S4.

**Figure 4:**
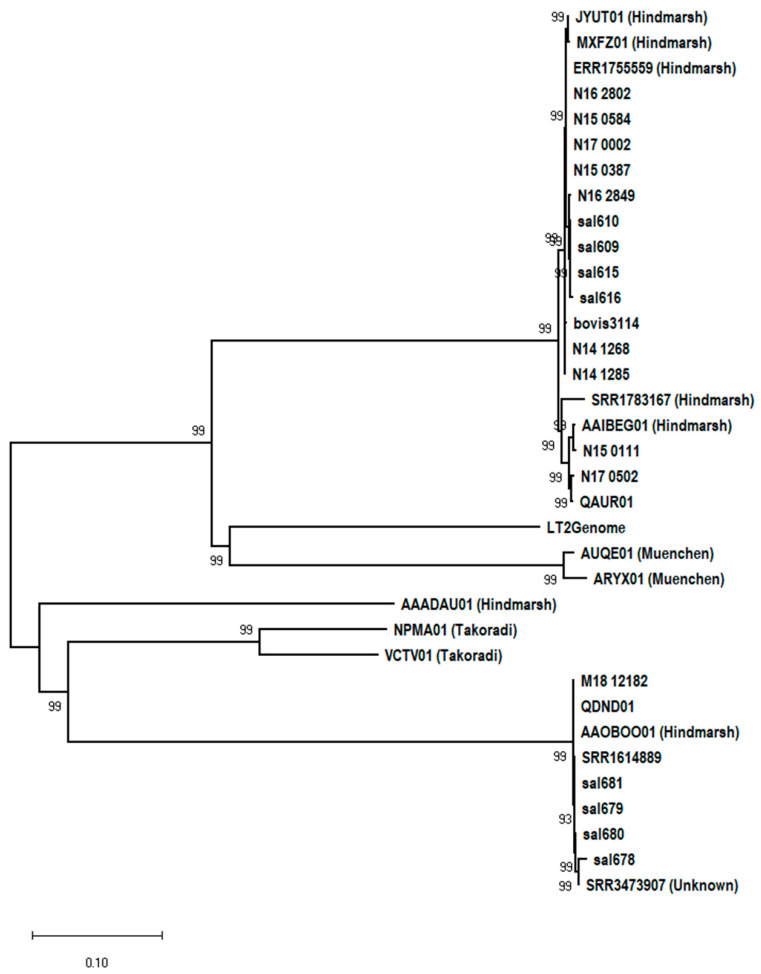
Phylogenomic profiling of *S.* Bovismorbificans and closely related serovars. *S*. Bovismorbificans is made up strains with two distinct genomic backbones, naturally grouped into two distant clusters. Cluster 1 strains are closer to *S*. Typhimurium (represented by the reference genome LT2) and *S.* Muenchen (AUQE01 and ARYX01) than to the Cluster 2 *S*. Bovismorbificans strains. WGS analysis from this study identified *S*. Takoradi (NPMA01 and VCTV01) as the closest serovar to the Cluster 2 *S.* Bovismorbificans strains. *S*. Hindmarsh strains sharing the antigen-cluster with *S.* Bovismorbificans in White–Kauffmann–Le Minor scheme also exhibit different genomic backbones as in the case of *S.* Bovismorbificans. Two of the Hindmarsh strains are aligned with Cluster 1 (SRR1783167 and AAIBEG01), while AAADAU01 and AAOBOO01 align within Cluster 2. For this analysis, Neighbor-Joining method implemented on MEGA X suite was used yielding a data matrix made up of 46,403 base positions across 35 genomes. MLST, DNA tiling Microarray (MA), whole genome sequence (WGS) based phylogenetic analysis, core-gene alleles spanning more than 2700 core genes, and k-mer binning based on NGS datasets and conventional assays all point to two divergent genome types bearing a single serotyping cluster. SRR3473907 was not clearly serotyped either by its NCBI BioSample record or by SeqSero in this study.

Other interesting findings were that a sample with assembly AAADAU01 was mistyped as *S.* Hindmarsh and appeared to be unrelated to any of the known *S.* Hindmarsh or *S.* Bovismorbificans strains. Furthermore, two ambiguously typed strains (AAOBOO01 and SRR3473907) with overlapping serotyping signatures were almost identical to the other ST150 *S.* Bovismorbificans strains. A subset of this data matrix was illustrated that depicts a snapshot of this whole genome-based *k-mer*-binning analysis (Figure 3) and the evolutionary distance between pair-wise genome comparisons is shown [represented by teal blue (highly similar) to sky blue (somewhat similar) to orange (highly dissimilar) values]. The evolutionary distance between pair-wise genome comparisons was shown in values from teal blue (highly similar) to sky blue (some similarity) to orange (highly dissimilar) interspersed with a gradient of tan values for decreasing similarity. Clusters 1 and 2 were placed apart and their individual comparisons shown in orange highlighted the evolutionary distance measured by the *k-mer*-binning method. Interestingly, *S.* Typhimurium LT2 and *S.* Muenchen AUQE01 shown in sky blue for comparison with Cluster 1 appeared to suggest a more similar genome backbone among these groups when compared with Cluster 2. Similarly, *S.* Takoradi strain (NPMA01) defined the boundary for Cluster 2 (shown in tan value) strains when compared with Cluster 1. A ST150 strain CIES13 appeared to have significant genomic divergence when compared with other Cluster 2 strains. When the WGS assemblies from these nearest neighbors of the two cluster groups were queried for wg-core SNP-based clustering (Figure 4), the patterns similar to the *k-mer* profiling, as observed in Figure 3, were observed. ST150 strains formed a separate cluster with two *S.* Takoradi strains as their nearest neighbors (Figure 4). AAADAU01, a mistyped *S.* Hindmarsh isolate, was identified as a strain of serovar *S.* Weltevreden based on its core-genome profile (data not shown). Two *S*. Muenchen strains and *S.* Typhimurium LT2 (reference genome) flanked the top Cluster 1 that contained *S.* Bovismorbificans genomes from this study and *S.* Hindmarsh strains from NCBI. The polyphyletic nature of *S.* Hindmarsh strains evident from our analysis needs further investigation. The wg-core gene method relied on identifying sequence variations (SNPs) in generating different clusters while the *k-mer* binning approach used the number of conserved *k-mers* between any two genomes. The concordance of the two different whole genome analytic methods we have applied to understand the phylogenetic relationship between the two clusters in this study was evident from these results illustrated in Figure 1, Figure 2, Figure 3 and Figure 4 and associated Supplemental File S4.

Intra-serovar differences among lineages within some *Salmonella* serovars are being recognized from the growing volume of WGS data from outbreak and surveillance samples, geographically distributed and within legacy strain collections. Genome-wide and core genome analysis of *Salmonella* serovars using WGS datasets has been proven to be capable of profiling underlying evolutionary processes contributing to the genome backbone differences among different lineages constituting a conventionally typed serovar [19,20,21,22,46,47]. The application of WGS technology for sequence-based serotyping [25,48], characterization of outbreak isolates [49,50], source-tracking in the epidemiological investigation of foodborne outbreaks, and phylogenetic relationships [51,52,53,54,55] have been well documented. The robust bioinformatic workflow from this study utilizes an ad hoc set of complete genome assemblies for high-resolution phylogenetic analysis and adds to the existing *S. enterica* core-genes (cgMLST) and whole genome MLST (wgMLST) typing schema hosted on public resources such as EnteroBase and SISTR [46,48]. Detailed genomic analysis in combination with sequence- and/or serology-based serotyping alone could provide a broader explanation for the complex intra-serovar differences that are not easily explained solely in terms of conventional serological methods.

### 3.5. Plasmids, Phage, AMR, and Virulence Profiling of S. Bovismorbificans Genome Types

**Figure 5:**
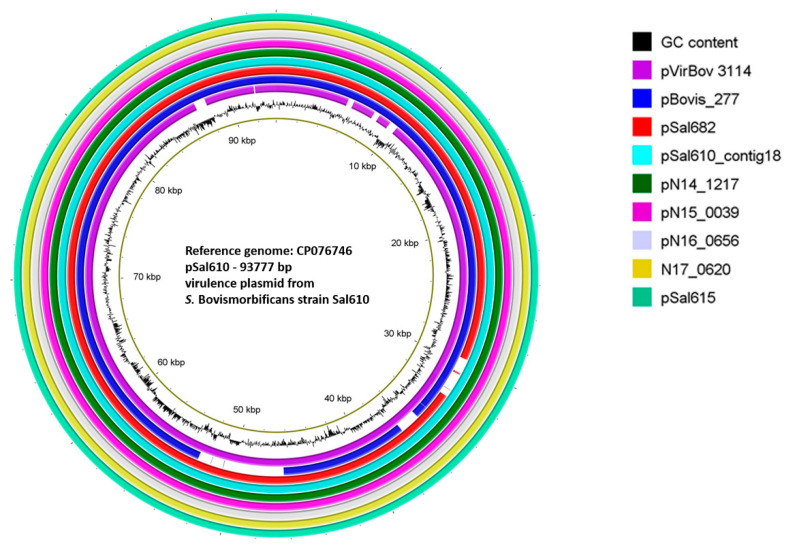
Comparison of pVirBov 3114, plasmids from Swiss strains and the closed assembly pSal610 (hummus outbreak). Closed genome of 93,777 bp long virulence plasmid from *S*. Bovismorbificans strain Sal610 (CP076746) was used as the reference genome to compare pVIRBov from strain 3114 [9] and a few selected Cluster 1 isolates (Table 1) from this study. BLAST+ based comparison and visualization were carried out on BRIG 0.95 [39]. White patches in a circle indicate any missing sequences in the query genome.

Predictive analysis of mobilome and virulence factor elements in the WGS assemblies of *S.* Bovismorbificans from this study was carried out on PlasmidFinder, PHASTER, SPIFinder and GalaxyTrakr AMRFinderPlus tools as described earlier. A 94 kb putative virulence plasmid, pVirBov (HF969016), such as the *S*. Typhimurium LT2 plasmid pSLT, was reported from a Malawian clinical *S.* Bovismorbificans strain 3114 [9]. PlasmidFinder web tool identified IncFIB(s), InfFII(s) plasmids in 59 out of 60 Cluster 1 strains (Supplemental Figure S3), while other plasmid types such as IncI1, IncI2, and colI56 were noted in a few strains (Supplemental File S5). Previously, the presence of a putative 90 kb plasmid was predicted [7] in some of the hummus-outbreak strains included in this study (Table 1). We designed a local BLAST analysis to identify the homologous sequences of this plasmid in the WGS assemblies. Fifty-seven *S.* Bovismorbificans strains hosted the homologous plasmid sequences on a single contig. The alignment of these contigs illustrated a possible loss of complete annotation (Supplemental File S4, mauve alignment of putative plasmid-bearing contigs). We sequenced the complete genome of the pVirBov-like virulence plasmid from the clinical strain Sal610. The 93,477 bp closed assembly of pSal610 was then used to compare with pVirBov and representative genome contigs from the study strains (Figure 5 and Supplemental Figure S4). Low complexity regions, missing sequences, and ambiguous sequence fragments in pVirBov, Bovis_277_contig24 and Sal682_contig35 were identified. The first complete sequence of *S.* Bovismorbificans virulence plasmid, pSal610 (CP076746), from clinical strain Sal610 isolated during a hummus outbreak in 2011, differed from the homologous pVirBov from strain 3114 by additional 360 bases. Further analysis is needed to understand the occurrence of this plasmid among other SE lineages, and its contribution to the virulence of the *S.* Bovismorbificans Cluster 1 strains.

**Figure 6:**
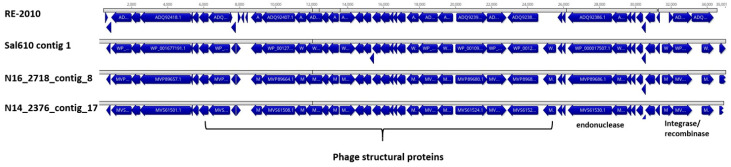
ST377 strains from clinical and food sources contain a unique *S*. Enteriditis RE-2010-like phage. An ELPhiS prophage RE-2010 (Accession: HM770079) from *S.* Enteriditis LK5 [59] was identified in ST377 genomes. A comparison of representative genomes from three strains isolated from different sample sources are illustrated above: Sal610 contig 1 (AZKX01000024)—Clinical, N16_2718_contig_8 (WSCE01000008)—Clinical and N14_2376_contig_17 (WSDY01000017)—Food, Onion. The *S.* Bovismorbificans version of the phage appeared to be homologous with predicted additional ORFs in the structure protein core. Prevalence of other phage sequences in *S.* Bovismorbificans strains from this study are listed in the Supplemental File S5.

PHASTER tool results (Supplemental File S5) suggested a variety of homologous sequences only in Cluster 1 strains. A *S*. Enteritidis ELPhiS prophage RE2010-like sequence was predicted only in ST377 strains from the 2011 hummus outbreak in Washington DC and in various isolates from the Swiss collection. A complete RE2010-like phage sequence was identified from the ST377 strains by BLAST analysis (data not shown). Figure 6 illustrates the presence of the RE2010-like phage in Sal610 (contig1), N16_2718 (contig8), and N14_2376 (contig 17). The GalaxyTrakr AMRFinderPlus tool was used to evaluate the presence of antimicrobial resistance genes that may be present in the 95 *S*. Bovismorbificans strains. Previously, genes conferring resistance to Quinolone and other antimicrobials had been reported in some serovars including *S*. Bovismorbificans [56,57]. The results presented in Table 2 show that seven Cluster 1 strains were identified to contain multiple resistance genes against antimicrobials, such as Cephalosporin, Quinolone, Tetracycline, Beta-Lactam, Tetracycline, Gentamicin, Kanamycin, Bleomycin, Bleomycin, Sulfonamide, Streptomycin, Chloramphenicol, Streptomycin and Quaternary Ammonium. The results of this predictive analysis (Supplemental File S3) further highlighted extensive genotypic differences between the two polyphyletic clusters identified in this study for *S.* Bovismorbificans and mirror the established pathogenic properties of major serovars such as Typhimurium, Enteritidis, Heidelberg, and Newport [58]. 

## 4. Conclusions

The high resolution WGS analyses of *S.* Bovismorbificans reported in this study and other SE serovars by others clearly highlight the need for a broader sequence-based genome pathotyping framework that recognizes genome-wide genetic boundaries (vertical) and the emergent adaptive/incidental features (horizontal) of the genome pathotypes even within conventionally defined serovars [20,21]. Together, these results demonstrate the genomic diversity of *S.* Bovismorbificans strains and provide details of the sequence diversity separating the two polyphyletic lineages recognized and characterized in this study. The schema of 2690 wg-core genome loci used to identify the conserved genome backbone could be applied to determine the genome structure of other SE serovars having intra-serovar differences and elucidate the genomic structure within a population of *S.* enterica pathogens that persist and circulate for years, with different STs succeeding over time. As demonstrated for *S.* Bovismorbificans, wg-core genome analysis could be used to identify misidentified strains by the limitations of conventional serotyping methods and ameliorate the characterization of emerging sub-groups within a serovar. The characteristic mobilome features described in this study will facilitate proper classification of emerging genome pathotypes of *S.* Bovismorbificans. The application of WGS datasets and methods based on the genome sequences have become an integral part of not just understanding the source-tracking of strains, but also in understanding the genomic diversity of *S. enterica* strains in food-safety investigations. The results from this study of *S.* Bovismorbificans using phylogenomic analysis highlight the need to use the WGS data, including annotations to expand our understanding of the genomic and phenotypic diversity among the populations of strains within all *Salmonella* serovars that display the potential to infect humans and in contaminating the animal food supply chain. A comprehensive understanding of emerging pathotypes also of minor serovars of *S. enterica* in addition to those already on top of the list for surveillance would necessarily complement ongoing post-genomics efforts to apply NGS data and methods to prevent new sources of foodborne illnesses and additional serovars adding to the growing list of *S. enterica* pathogens.

## Figures and Tables

**Table 1:** Strain information (Strain name, Source, Country, Year of isolation, and Reference), genomic characterization (Genome size, No. of CDSs, and Sequence Type), NCBI Biosample, and GenBank accession numbers of 95 *S.* Bovismorbificans isolates used in this study.

Strain	Source	Country	Year	Genome Size (kb)	No. of CDSs	ST ^a^	NCBI Biosample	NCBI Accession No.	Reference
M18_12182	Environment	USA	2018	4679	4399	150	SAMN12657258	WSCV00000000	This study
Bovis_38	Beef	Canada	1989	4579	4321	142	SAMN12657217	WSCW00000000	This study
Bovis_120	Seafood, squid	Canada	missing	4706	4474	1499	SAMN12657218	WSCX00000000	This study
Bovis_187	Seafood, shrimp	Canada	1989	4739	4498	1499	SAMN12657204	WSCY00000000	This study
Bovis_277	Clinical	Canada	1984	4832	4638	1499	SAMN12657210	WSCZ00000000	This study
Bovis_278	Clinical	Canada	1984	4738	4498	142	SAMN12657232	WSDA00000000	This study
N14_0147	Clinical	Switzerland	2014	4690	4471	142	SAMN12657231	WSDB00000000	This study
N14_0646	Clinical	Switzerland	2014	4702	4449	1499	SAMN12657228	WSDC00000000	This study
N14_1189	Clinical	Switzerland	2014	4790	4575	142	SAMN12657257	WSDD00000000	This study
N14_1190	Clinical	Switzerland	2014	4784	4566	142	SAMN12657229	WSDE00000000	This study
N14_1194	Clinical	Switzerland	2014	4789	4580	142	SAMN12657219	WSDF00000000	This study
N14_1212	Clinical	Switzerland	2014	4790	4577	142	SAMN12657254	WSDG00000000	This study
N14_1217	Clinical	Switzerland	2014	4854	4688	142	SAMN12657212	WSDH00000000	This study
N14_1222	Clinical	Switzerland	2014	4791	4577	142	SAMN12657214	WSDI00000000	This study
N14_1232	Clinical	Switzerland	2014	4778	4560	142	SAMN12657205	WSDJ00000000	This study
N14_1233	Clinical	Switzerland	2014	4790	4568	142	SAMN12657209	WSDK00000000	This study
N14_1237	Clinical	Switzerland	2014	4781	4565	142	SAMN12657230	WSDL00000000	This study
N14_1238	Clinical	Switzerland	2014	4781	4563	142	SAMN12657235	WSDM00000000	This study
N14_1239	Clinical	Switzerland	2014	4778	4562	142	SAMN12657165	WSDN00000000	This study
N14_1252	Clinical	Switzerland	2014	4781	4562	142	SAMN12657181	WSDO00000000	This study
N14_1255	Clinical	Switzerland	2014	4786	4576	142	SAMN12657154	WSDP00000000	This study
N14_1266	Clinical	Switzerland	2014	4779	4561	142	SAMN12657194	WSDQ00000000	This study
N14_1267	Clinical	Switzerland	2014	4775	4602	142	SAMN12657197	WSDR00000000	This study
N14_1268	Clinical	Switzerland	2014	4783	4567	142	SAMN12657192	WSDS00000000	This study
N14_1285	Clinical	Switzerland	2014	4780	4561	142	SAMN12657190	WSDT00000000	This study
N14_1527	Clinical	Switzerland	2014	4813	4590	1499	SAMN12657150	WSDU00000000	This study
N14_1673	Clinical	Switzerland	2014	4819	4609	142	SAMN12657188	WSDV00000000	This study
N14_1674	Clinical, blood	Switzerland	2014	4783	4571	142	SAMN12657156	WSDW00000000	This study
N14_1962	Clinical	Switzerland	2014	4715	4468	1499	SAMN12657164	WSDX00000000	This study
N14_2376	Food, onion	Switzerland	2014	4723	4486	377	SAMN12657147	WSDY00000000	This study
N15_0039	Food, grain	Switzerland	2015	4726	4493	377	SAMN12657185	WSDZ00000000	This study
N15_0111	Clinical	Switzerland	2015	5024	4860	1499	SAMN12657157	WSEA00000000	This study
N15_0387	Clinical	Switzerland	2015	4643	4404	142	SAMN12657198	WSEB00000000	This study
N15_0584	Clinical	Switzerland	2015	4670	4468	142	SAMN12657182	WSEC00000000	This study
N15_1010	Food	Switzerland	2015	4725	4483	377	SAMN12657196	WSED00000000	This study
N15_1120	Food	Switzerland	2015	4724	4486	377	SAMN12657193	WSEE00000000	This study
N15_1199	Food	Switzerland	2015	4720	4485	377	SAMN12657191	WSEF00000000	This study
N15_1496	Clinical	Switzerland	2015	4738	4530	142	SAMN12657189	WSEG00000000	This study
N15_2048	Clinical	Switzerland	2015	4847	4671	1499	SAMN12657202	WSEH00000000	This study
N15_2285	Clinical	Switzerland	2015	4806	4598	377	SAMN12657199	WSEI00000000	This study
N15_2358	Clinical	Switzerland	2015	4893	4724	377	SAMN12657233	WSEJ00000000	This study
N16_0208	Clinical	Switzerland	2016	4798	4571	1499	SAMN12657155	WSBH00000000	This study
N16_0447	Clinical, urine	Switzerland	2016	4636	4399	142	SAMN12657160	WSBI00000000	This study
N16_0656	Clinical	Switzerland	2016	4838	4628	2640	SAMN12657256	WSBJ00000000	This study
N16_1158	Clinical	Switzerland	2016	4781	4566	142	SAMN12657253	WSBK00000000	This study
N16_1250	Clinical	Switzerland	2016	4788	4571	142	SAMN12657211	WSBL00000000	This study
N16_1558	Clinical	Switzerland	2016	4571	4290	150	SAMN12657220	WSBM00000000	This study
N16_1675	Clinical	Switzerland	2016	4782	4573	142	SAMN12657207	WSBN00000000	This study
N16_1715	Clinical	Switzerland	2016	4784	4570	142	SAMN12657200	WSBO00000000	This study
N16_1722	Clinical	Switzerland	2016	4785	4569	142	SAMN12657206	WSBP00000000	This study
N16_1740	Clinical	Switzerland	2016	4778	4556	142	SAMN12657234	WSBQ00000000	This study
N16_1757	Clinical	Switzerland	2016	4784	4568	142	SAMN12657203	WSBR00000000	This study
N16_1786	Clinical	Switzerland	2016	4781	4567	142	SAMN12657259	WSBS00000000	This study
N16_1788	Clinical	Switzerland	2016	4784	4584	142	SAMN12657260	WSBT00000000	This study
N16_1794	Clinical	Switzerland	2016	4794	4573	142	SAMN12657221	WSBU00000000	This study
N16_1829	Clinical	Switzerland	2016	4785	4571	142	SAMN12657255	WSBV00000000	This study
N16_2042	Clinical	Switzerland	2016	4778	4561	142	SAMN12657208	WSBW00000000	This study
N16_2132	Clinical	Switzerland	2016	4787	4563	142	SAMN12657216	WSBX00000000	This study
N16_2207	Animal, cat	Switzerland	2016	4778	4573	142	SAMN12657146	WSBY00000000	This study
N16_2424	Clinical	Switzerland	2016	4775	4556	142	SAMN12657163	WSBZ00000000	This study
N16_2574	Clinical	Switzerland	2016	4947	4754	142	SAMN12657148	WSCA00000000	This study
N16_2598	Clinical	Switzerland	2016	4706	4481	142	SAMN12657161	WSCB00000000	This study
N16_2682	Clinical	Switzerland	2016	4777	4574	142	SAMN12657159	WSCC00000000	This study
N16_2712	Clinical	Switzerland	2016	4703	4489	142	SAMN12657184	WSCD00000000	This study
N16_2718	Clinical	Switzerland	2016	4723	4482	377	SAMN12657144	WSCE00000000	This study
N16_2802	Clinical	Switzerland	2016	4724	4508	142	SAMN12657195	WSCF00000000	This study
N16_2849	Clinical	Switzerland	2016	4725	4489	377	SAMN12657142	WSCG00000000	This study
N16_2955	Clinical	Switzerland	2016	4811	4599	1499	SAMN12657187	WSCH00000000	This study
N17_0002	Clinical	Switzerland	2017	4706	4492	142	SAMN12657149	WSCI00000000	This study
N17_0502	Clinical	Switzerland	2017	4873	4696	1499	SAMN12657152	WSCJ00000000	This study
N17_0607	Clinical	Switzerland	2017	4640	4401	142	SAMN12657145	WSCK00000000	This study
N17_0620	Food	Switzerland	2017	4731	4493	377	SAMN12657151	WSCL00000000	This study
N17_0857	Feed	Switzerland	2017	4724	4507	377	SAMN12657158	WSCM00000000	This study
N17_1212	Clinical	Switzerland	2017	4773	4576	2640	SAMN12657162	WSCN00000000	This study
N17_1364	Clinical	Switzerland	2017	4869	4680	2640	SAMN12657186	WSCO00000000	This study
N17_1393	Clinical	Switzerland	2017	4779	4564	2640	SAMN12657153	WSCP00000000	This study
N17_2111	Clinical	Switzerland	2017	4775	4561	2640	SAMN12657143	WSCQ00000000	This study
N18_0002	Clinical	Switzerland	2018	4805	4605	142	SAMN12657183	WSCR00000000	This study
N18_0969	Clinical	Switzerland	2018	4640	4402	142	SAMN12657213	WSCS00000000	This study
N18_1092	Clinical	Switzerland	2018	4728	4492	377	SAMN12657215	WSCT00000000	This study
N18_1144	Clinical	Switzerland	2018	4722	4544	377	SAMN12657201	WSCU00000000	This study
Sal609 ^b^	Clinical	USA	2011	4896	4925	377	SAMN02422699	AZKW00000000	[11]
Sal610	Clinical	USA	2011	4857	4870	377	SAMN02422700	AZKX00000000	[11]
Sal615	Clinical	USA	2011	4845	4891	377	SAMN02422701	AZKY00000000	[11]
Sal616	Food	USA	2011	4865	4893	377	SAMN02422702	AZKZ00000000	[11]
Sal617	Food	USA	2011	4872	4887	377	SAMN02422703	AZLA00000000	[11]
Sal644	Clinical	USA	2001	4769	4769	1499	SAMN02422688	AZLC00000000	[11]
Sal676	Clinical	USA	2012	4569	4476	150	SAMN02422698	AZKV00000000	[11]
Sal677	Clinical	USA	2012	4663	4614	150	SAMN02422693	AZKR00000000	[14]
Sal678	Clinical	USA	2012	4579	4629	150	SAMN02422694	AZKS00000000	[14]
Sal679	Clinical	USA	2012	4567	4471	150	SAMN02422695	AZKT00000000	[14]
Sal680	Clinical	USA	2012	4596	4506	150	SAMN02422696	AZKU00000000	[14]
Sal681	Clinical	USA	2012	4575	4492	150	SAMN02422697	AZLB00000000	[14]
Sal682	Clinical	USA	2012	4926	4944	377	SAMN02422690	AZKQ00000000	[11]
Sal683	Clinical	USA	2012	4574	4586	150	SAMN02422689	AZKP00000000	[14]
bovis3114 ^b^	Clinical	Malawi	1997	4680	4599	142	SAMEA3138815	HF969015	[9]
bovispt13	Unknown	Unknown	Unknown	NA ^c^	NA	Unknown ^d^	SAMN01081634	SRS347148 ^e^	NCBI SRA
boviscies13	Water	Mexico	2013	NA	NA	150	SAMN02335370	SRS476367 ^f^	NCBI SRA
pSal610	Clinical	USA	2011	93.8	111	377	SAMN02422700	CP076746	This study

^a^ Sequence type (ST) was determined by uploading genome assemblies to https://cge.cbs.dtu.dk/services/MLST/ (accessed on 1 June 2020). ^b^ Genome size and number of CDSs of strains named with ‘Sal’ and ‘bovis’ were determined by the SEED Viewer of RAST annotation. ^c^ NA represents ‘not available’. ^d^ Nearest ST is 142. ^e,f^ Public sequence reads were downloaded from NCBI SRA (Sequence Read Archive) and locally assembled. Note: External genome sequences downloaded from NCBI were downloaded by entering the assembly accessions at the end of the URL: https://www.ncbi.nlm.nih.gov/nuccore?term= (last accessed on 6 April 2022) QDND01, MXTS01, MZFY01, QAUR01, MXXQ01, MYFY01, WFIN01, MXTX01, MXUD01, JYUT01, MXFZ01, QAUR01, AUQE01, ARYX01, AAADAU000000000.1 (for AAADAU01), NPMA01, VCTV01, AAOBOO000000000.1 (for AAOBOO01), AAIBEG000000000.1 (for AAIBEG01). Four sequence reads datasets were downloaded from NCBI SRA database: ERR1755559, SRR1783167, SRR16148890 and SRR3473097 for generating WGS assemblies for this analysis.

**Table 2:** Antimicrobial resistance genes identified in the *S.* Bovismorbificans genomes ^a^.

Strain	Gene	Subclass	Sequence Name/Description
N14_0147	*aph(3* *″)-Ib*	Streptomycin	Aminoglycoside O-phosphotransferase APH(3″)-Ib
	*sul2*	Sulfonamide	Sulfonamide-resistant dihydropteroate synthase Sul2
	*aph(6)-Id*	Streptomycin	Aminoglycoside O-phosphotransferase APH(6)-Id
N15_0111	*bla_CTX-M-55_*	Cephalosporin	Class A extended-spectrum beta-lactamase CTX-M-55
	*qnrS1*	Quinolone	Quinolone resistance pentapeptide repeat protein QnrS1
	*tet(A)*	Tetracycline	Tetracycline efflux MFS transporter Tet(A)
	*blaTEM-1*	Beta-lactam	Class A broad-spectrum beta-lactamase TEM-1
	*tet(M)*	Tetracycline	Tetracycline resistance ribosomal protection protein Tet(M)
	*aac(3)-IId*	Gentamicin	Aminoglycoside N-acetyltransferase AAC(3)-IId
	*aph(3′)-IIa*	Kanamycin	Aminoglycoside O-phosphotransferase APH(3′)-IIa
	*ble*	Bleomycin	Bleomycin binding protein BLMT
	*bleO*	Bleomycin	Bleomycin binding protein
	*sul3*	Sulfonamide	Sulfonamide-resistant dihydropteroate synthase Sul3
	*aadA2*	Streptomycin	ANT(3″)-Ia family aminoglycoside Nucleotidyltransferase AadA2
	*cmlA1*	Chloramphenicol	Chloramphenicol efflux MFS transporter CmlA1
	*aadA1*	Streptomycin	ANT(3″)-Ia family aminoglycoside Nucleotidyltransferase AadA1
	*qacL*	Quaternary ammonium ^b^	Quaternary ammonium compound efflux SMR transporter QacL
N16_0208	*aph(6)-Id*	Streptomycin	Aminoglycoside O-phosphotransferase APH(6)-Id
	*sul2*	Sulfonamide	Sulfonamide-resistant dihydropteroate synthase Sul2
	*aph(3* *″)-Ib*	Streptomycin	Aminoglycoside O-phosphotransferase APH(3″)-Ib
	*tet(A)*	Tetracycline	Tetracycline efflux MFS transporter Tet(A)
N16_2574	*dfrA1*	Trimethoprim	Trimethoprim-resistant dihydrofolate reductase DfrA1
	*bla_TEM-1_*	Beta-lactam	Class A broad-spectrum beta-lactamase TEM-1
	*sul2*	Sulfonamide	Sulfonamide-resistant dihydropteroate synthase Sul2
	*bla_CTX-M-1_*	Cephalosporin	Class A extended-spectrum beta-lactamase CTX-M-1
N16_2598	*tet(A)*	Tetracycline	Tetracycline efflux MFS transporter Tet(A)
N16_2955	*aph(3* *″)-Ib*	Streptomycin	Aminoglycoside O-phosphotransferase APH(3″)-Ib
	*aph(6)-Id*	Streptomycin	Aminoglycoside O-phosphotransferase APH(6)-Id
	*tet(A)*	Tetracycline	Tetracycline efflux MFS transporter Tet(A)
	*sul2*	Sulfonamide	Sulfonamide-resistant dihydropteroate synthase Sul2
N17_0502	*floR*	Chloramphenicol/florfenicol	Chloramphenicol/florfenicol efflux MFS transporter FloR
	*qnrB19*	Quinolone	Quinolone resistance pentapeptide repeat protein QnrB19
	*sul2*	Sulfonamide	Sulfonamide-resistant dihydropteroate synthase Sul2
	*aph(3″)-Ib*	Streptomycin	Aminoglycoside O-phosphotransferase APH(3″)-Ib
	*aph(6)-Id*	Streptomycin	Aminoglycoside O-phosphotransferase APH(6)-Id
	*tet(A)*	Tetracycline	Tetracycline efflux MFS transporter Tet(A)

^a^ Galaxy GenomeTrakr AMRFinder tool used for identification of antimicrobial resistance (AMR). All FASTA genomes of 98 *S.* Bovismorbificans were scanned, and 7 strains captured the acquired antimicrobial resistance genes. ^b^ Element type: Stress; Element subtype: Biocide.

## Data Availability

All WGS datasets have been submitted to the NCBI GenomeTrakr project and the details are provided in Table 1. Supplemental Files S1–S6, Supplemental Figures S1–S4, and Supplemental Table S1 contain additional information and illustrations relevant to the results presented in the main text.

## Supplementary Materials

The following supporting information can be downloaded at: https://www.mdpi.com/article/10.3390/microorganisms10061199/s1, Supplemental File S1 to S6 consisting of the list of serovars used to develop wg-core genome scheme, Jaccard matrix comparing 330 genomes using *k-mers*, mobilome data, 2650 core genes developed as part of this study, and a large SNP matrix consisting of 48,344 positions in 2512 of 2650 core genes; Supplemental Figures S1A,B and S2–S4 with data mining of microarray analysis of *S.* Bovismorbificans from hummus, other foods, clinical and unknown origins, and a snapshot of MAUVE comparison of the pSal610 virulence plasmid with many other representative strains; and Supplemental Table S1 with allelic profiles by 6 ST spanning all the known *S.* Bovismorbificans isolates.
