# Supplementary material for: Phylogenomic Analysis of Salmonella enterica subsp. enterica Serovar Bovismorbificans from Clinical and Food Samples Using Whole Genome Wide Core Genes and kmer Binning Methods to Identify Two Distinct Polyphyletic Genome Pathotypes
Source: Microorganisms. 2022 Jun 11;10(6):1199. doi: 10.3390/microorganisms10061199 (PMC9228720; doi:10.3390/microorganisms10061199)
Supplement: Supplementary file 1 [file microorganisms-10-01199-s001.zip › Supplemental_Files_Figures.pdf]

List of Supplemental Files in MS Excel format

Supplemental_File_S1
Final list of GenBank Accessions for 645 complete genomes from 150 *Salmonella* serovars. After initial BLAST analysis, 150 representative closed genomes were used to define 2650 core genes based on LT2 gene sequences.

Supplemental_File_S2
SNP Matrix Generated from *S.* Bovismorbificans isolates from this study. [12 MB CSV file machine-readable or could be opened in MS Excel and is also available online due to its size at: https://github.com/gopal-gopinath/S.bovismorbificans-SNP-matrix1] Core genes and the SNP matrix generated when querying a database of 120 isolates are given. Each row consists of SNPs in a single base position of the core gene based on LT2 genome sequences. For most of the loci, more than one SNP is reported. Total number of rows (= bases) with SNPs is 48,344 spanning 2512 of 2650 core genes in the schema used for this study.

Supplemental_File_S3
Emerging properties of *S.* Bovismorbificans strains: Single Nucleotide Polymorphism (SNPs) in genomes of STs: 377,1499,2460 and 150 when compared with ST142 are given with core gene, chromosomal position, gene Position and annotated product encoded by the core gene locus.

Supplemental_File_S4
Jaccard matrix generated using *k-mer* comparisons of 330 genomes from the serovars *S.* Bovismorbificans Hindmarsh, Muenchen and Takoradi (total =330 X 330 comparative matrix). Heatmap red to green = most divergent to most similar

Supplemental_File_S5
Mobilome data. SPIFinder and PlasmidFinder results are in the worksheet 1. Presence of pSal610 in the isolates are also listed. Worksheet 2 has data from PHASTER.

Supplemental_File_S6
2650 core genes developed for this study using 645 complete genomes and 80+ *S*. Bovismorbificans WGS assemblies are listed. The IDs, genome coordinates and annotations are based on the LT2 genome (# NC_003197) from GenBank.

Supplemental_Figure_S1A:. Initial microarray analysis (MA) of *S.* Bovismorbificans strains obtained from · mmus outbreak and surveillance efforts

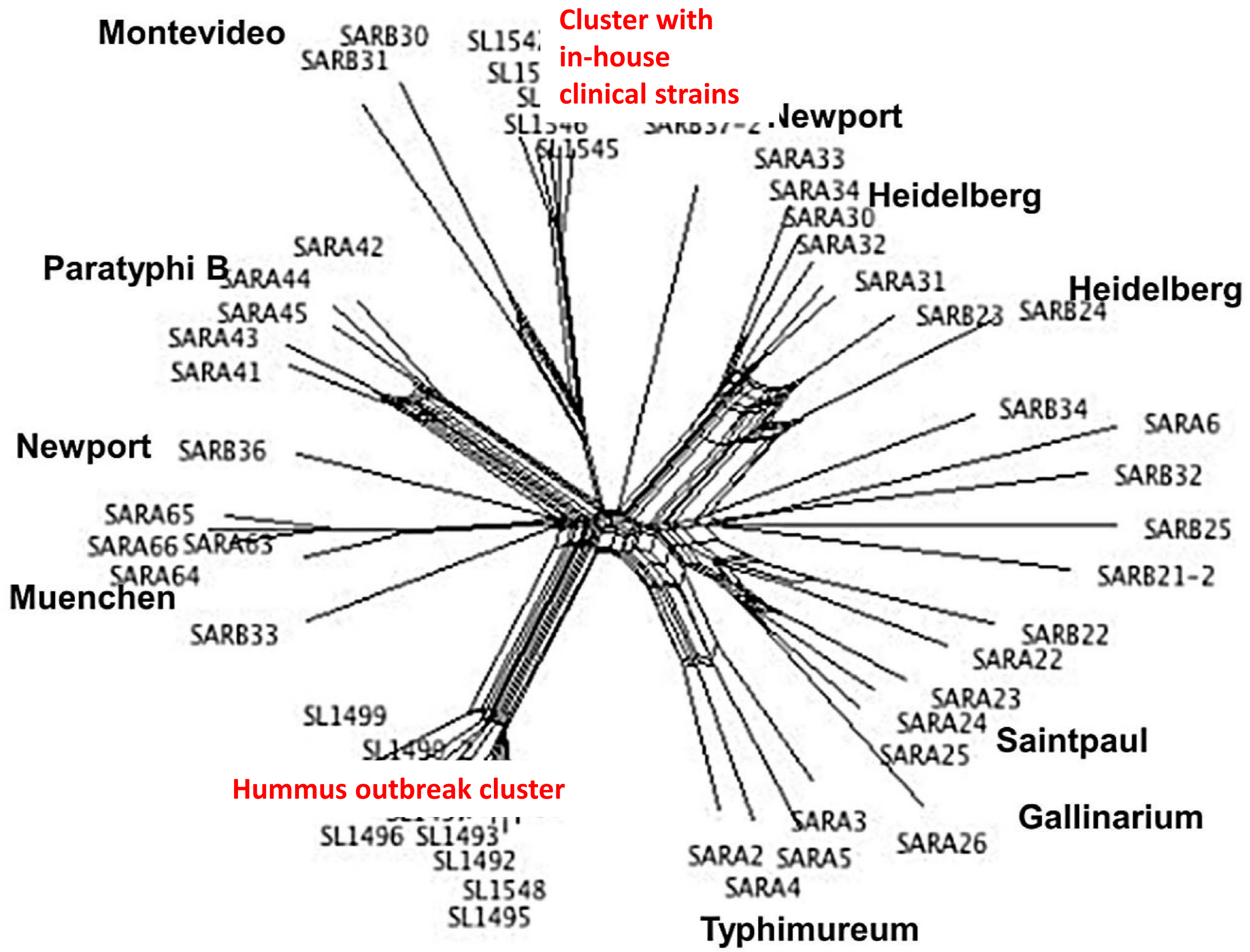

In this approach, a query isolate DNA is hybridized to the microarray chip the resulting hybridization data matrix from every query gets algorithmically compared with the digital profiles of thousands of SE isolates. Unlike conventional techniques, this platform applies sequence probes from annotated *Salmonella* genomes, phage and plasmids that provide a uniform platform to capture differences in the genomic backbone and known mobilome of queried strains

This platform consists a custom DNA tiling array consisting of 96,000 probes (1) used for high resolution genotyping of *Salmonella* serovars. For this analysis digital microarray profiles of legacy and in-house surveillance collections were chosen

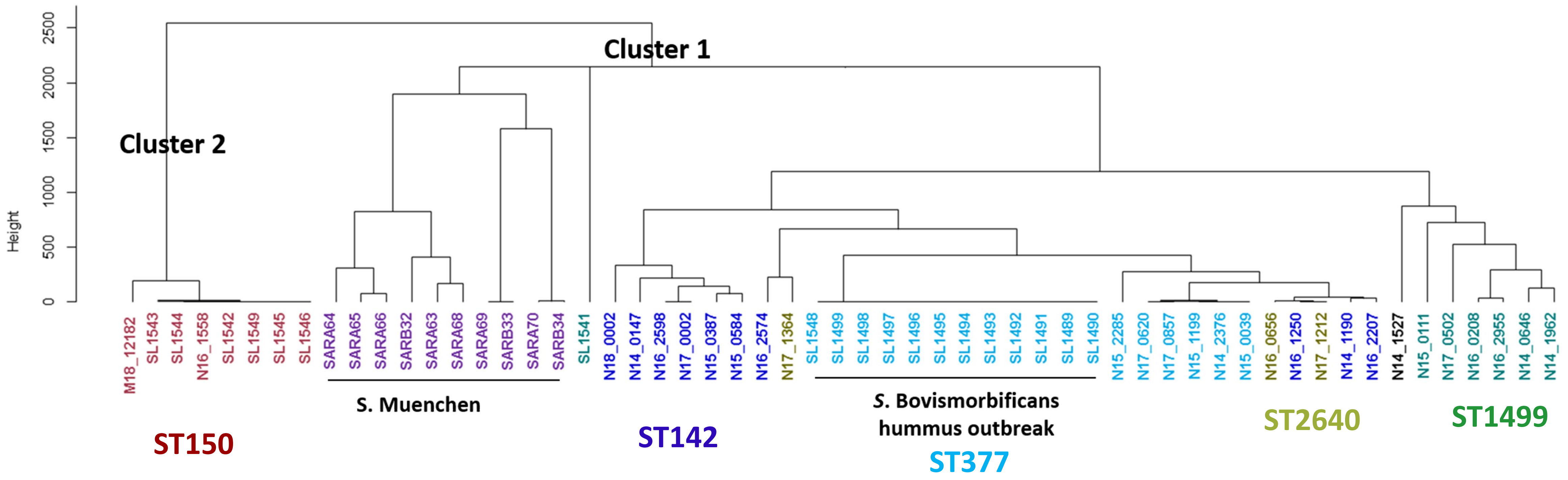

Supplemental_Figure_S1B: **Microarray analysis (MA) of *S.* Bovismorbificans strains obtained from clinical, food and unknown origins.**

Thirty *S.* Bovismorbificans were evaluated as described by Li *et al.* (2016) using the SEEC DNA microarray, a custom Affymetrix array that was developed with genomes of 38 *Salmonella enterica* strains. The SEEC microarray database annotations for the strains used in this study are given in parenthesis: Sal609 (SL1489), Sal610 (SL1490), Sal611 (SL1491), Sal612 (SL1492), Sal613 (SL1493), Sal614 (SL1494), Sal615 (SL1495), Sal616 (SL1496), Sal617 (SL1497), Sal618 (SL1498), Sal619 (SL1499), Sal676 (SL1542), Sal677 (SL1543), Sal678 (SL1544), Sal679 (SL1545), Sal680 (SL1546), Sal681 (SL1547), Sal682 (SL1548), Sal683 (SL1549), Sal644 (SL1541). The digital profiles SARA and SARB strains shown above were obtained from the MA database and were based on the collection reported in Achtman *et al.* (2013): Muenchen – IP6/88 (SARA63), NVSL519 (SARA64), NVSL2817 (SARA64), CDCB2026 (SARA66), IP15/88 (SARA68), IP11/88 (SARA69), IP25/88 (SARA66), ATCC8388 (SARB32), IP11/88 (SARB33) and IP25/88 (SARB34). Color code: Maroon: ST150; Purple: S. Muenchen; Green: S. Newport; Dark Blue: ST142; Light Blue: ST377; Olive Green:ST2640 and Teal: ST1499. Refer to Table 1 for specific strains – ST determination.

Supplemental_Figure_S2

This is the vertical version of the circular tree illustrated in Figure 1 of the results. This annotated image is zoomable for clarity. Additionally, the original TIFF image of the vertical tree is also provided in the GitHub folder: https://github.com/gopal-gopinath/S.bovismorbificans-SNP-matrix1

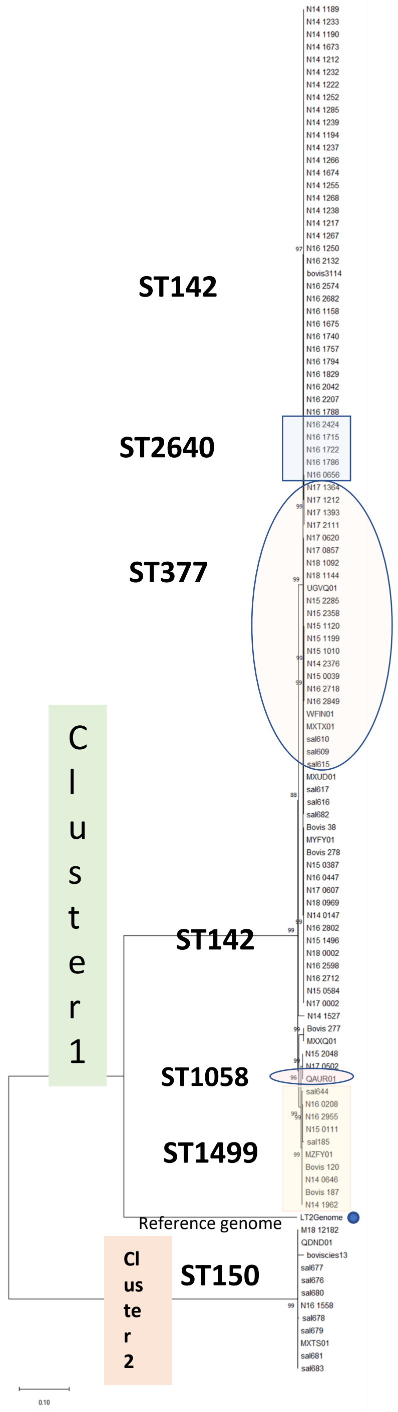

Plasmid replicon abundance in *S*. Bovismorbificans isolates from this study

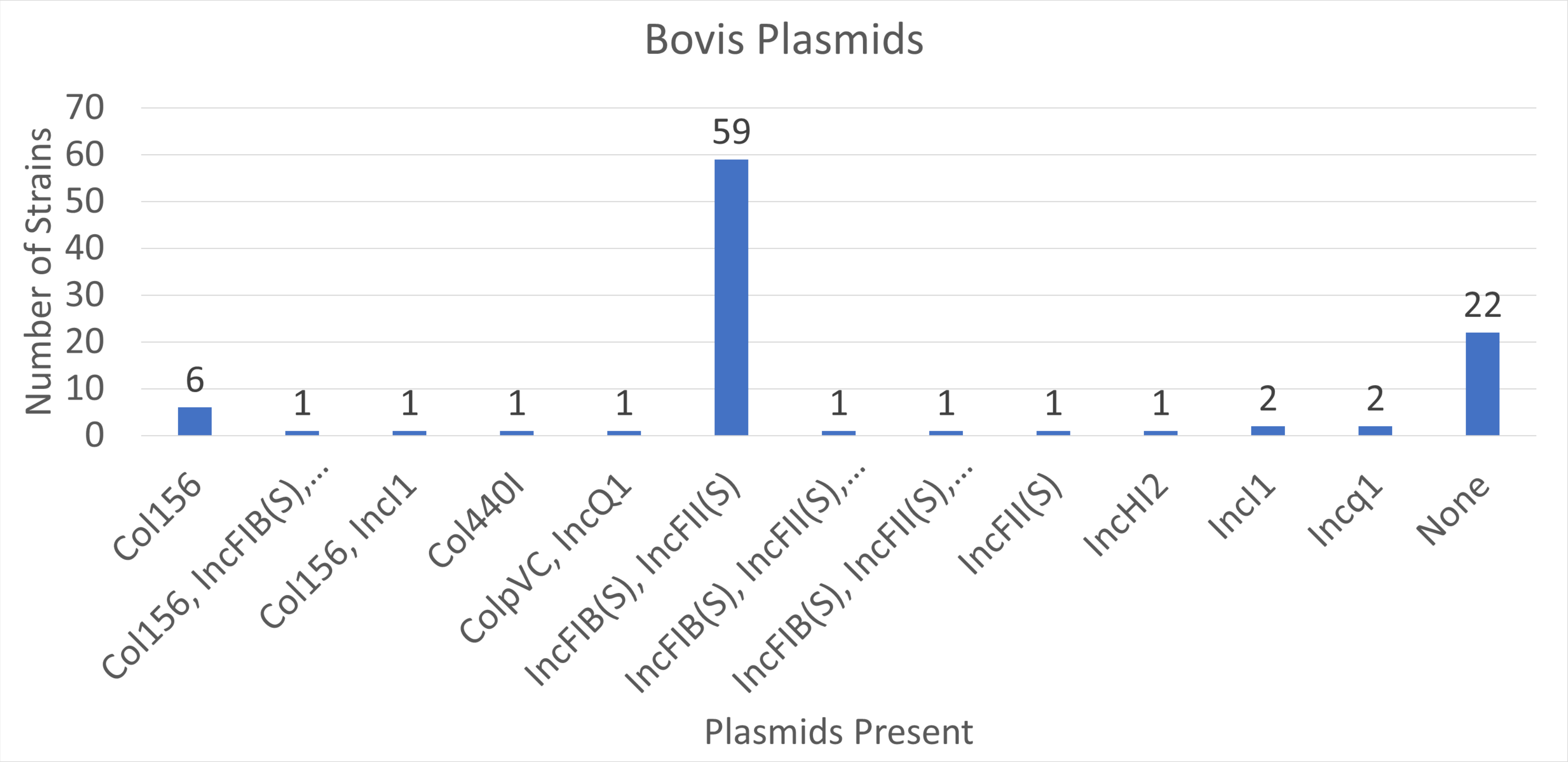

Supplemental_Figure_S3

Shown are the number of times the indicated replicon was identified within the isolates using the PlasmidFinder (2) database (https://cge.cbs.dtu.dk/services/PlasmidFinder/) and an 80% nucleotide identity cutoff threshold

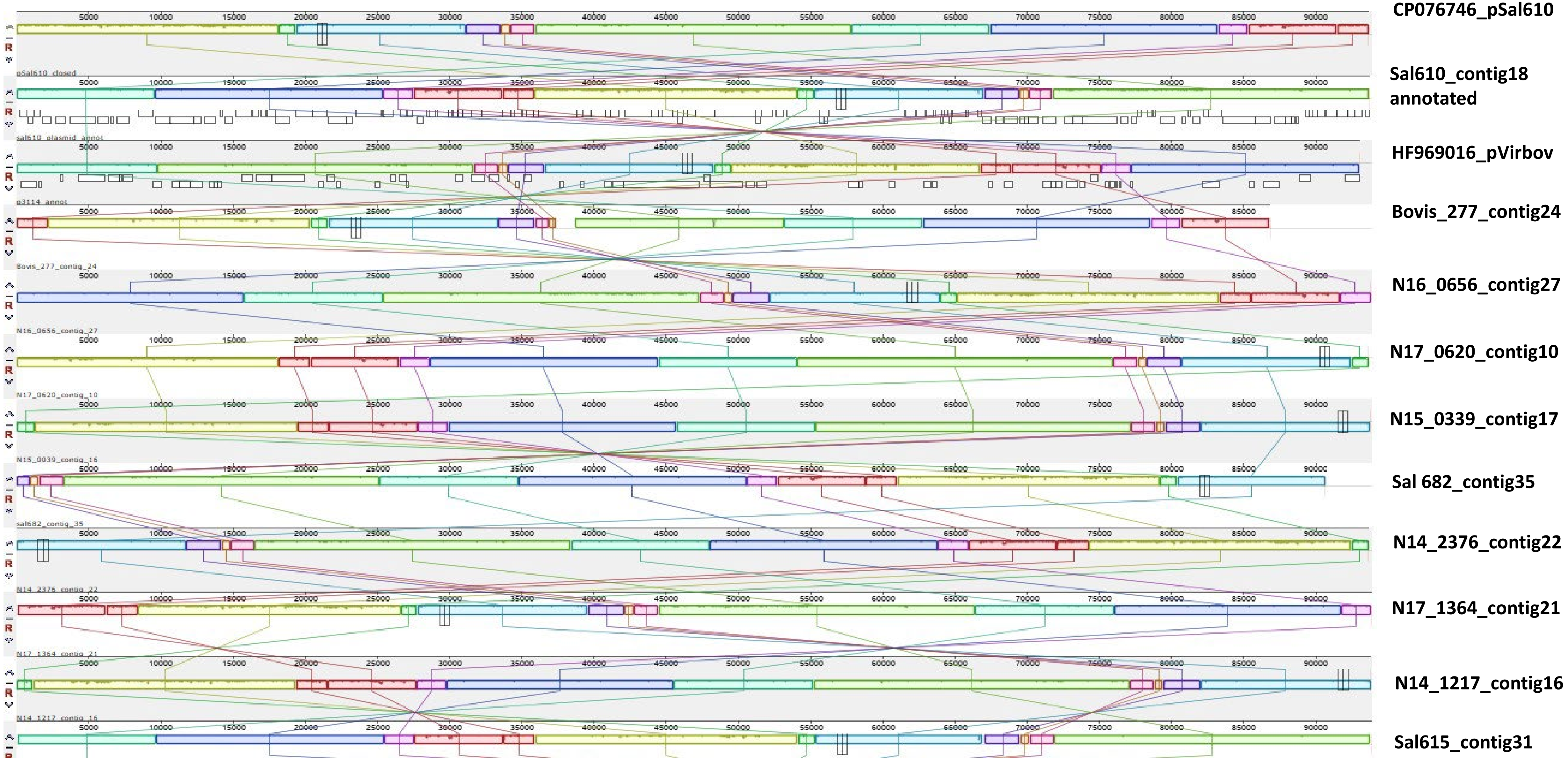

Supplemental_Figure_S4 - **Conserved virulence plasmid in 59 *S*. Bovismorbificans strains (refer Table 1) in Cluster 1 from this study.**

In this analysis, mauve alignment of a 93 Kb virulence plasmid from representative food, feed and clinical strains are shown. 93,377 bp long closed plasmid genome from strain Sal610 (GenBank Accession: CP076746) was used to anchor contigs containing plasmid sequences in various qualities of assembly.

ST	*aroC*	*dnaN*	*hemD*	*hisD*	*purE*	*sucA*	*thrA*
**377**	2	59	23	64	38	61	122
**142**	2	59	23	64	38	61	12
**1499**	2	59	23	64	38	19	12
**2640**	2	513	23	64	38	61	12
**1058**	2	59	254	64	38	19	12
**150**	61	12	10	65	54	63	57

Supplemental_Table_S1: Allelic profiles defined by 6 STs spanning all the known *S.* Bovismorbificans isolates

ST designations were assigned using MLST scheme of Center for Genomic Epidemiology (CGE) (http://cge.cbs.dtu.dk/services/MLST, last accessed 1/8/2020). based on seven housekeeping gene sequences (*aroC, dnaN, hemD, hisD, purE, sucA, and thrA*) (**3**)

References used in the Supplemental Files:

1. Li B, Jackson S, Gangiredla J, Wang W, Liua H, Tall BD, Jean-Gilles Beaubrun J, Jay-Russell M, Vellidis G, and Elkins CA. 2015. Genomic Evidence Reveals Numerous Re-Introduction Events of *Salmonella enterica* serovar Newport in Irrigation Ponds in Suwannee Watershed. Appl Environ Microbiol. 81: 8243-8253

2. Carattoli A, Zankari E, García-Fernández A, Voldby Larsen M, Lund O, Villa L, Møller Aarestrup F, Hasman H.

In silico detection and typing of plasmids using PlasmidFinder and plasmid multilocus sequence typing.

Antimicrob Agents Chemother. 2014 Jul;58(7):3895-903.

3. Achtman M, Wain J, Weill FX, Nair S, Zhou Z, Sangal V, Krauland MG, Hale JL, Harbottle H, Uesbeck A, Dougan G, Harrison LH, Brisse S; S. Enterica MLST Study Group. Multilocus sequence typing as a replacement for serotyping in *Salmonella* enterica. PLoS Pathog. 2012;8(6):e1002776.
